# Whole-Genome Sequence of a Beak and Feather Disease Virus Isolate from a Fledgling Red-Capped Parrot (*Purpureicephalus spurius*)

**DOI:** 10.1128/genomeA.01108-16

**Published:** 2016-10-13

**Authors:** Shubhagata Das, Sarker Subir, Jade K. Forwood, Seyed A. Ghorashi, Shane R. Raidal

**Affiliations:** a School of Animal and Veterinary Sciences, Charles Sturt University, Wagga Wagga, New South Wales, Australia; b Department of Physiology, Anatomy and Microbiology, School of Life Sciences, La Trobe University, Melbourne, Victoria, Australia; c School of Biomedical Sciences, Charles Sturt University, Wagga Wagga, New South Wales, Australia

## Abstract

The complete genome sequence of beak and feather disease virus (BFDV) from a fledgling red-capped parrot (*Purpureicephalus spurius*) was assembled and characterized. The genome consists of 1,995 nucleotides and encodes two major proteins in opposing directions. This is the first evidence of BFDV infectivity and a complete genome sequence for this novel host.

## GENOME ANNOUNCEMENT

Beak and feather disease virus (BFDV) is a single-stranded DNA (ssDNA) virus from the *Circoviridae* family and a globally distributed pathogen for the *Psittaciformes* birds causing psittacine beak and feather disease (PBFD) (1–4). The genome is highly compact, ambisense, and only approximately 2,000 nucleotides long; it encodes two major bidirectionally transcribed proteins, known as replication-initiator protein (Rep) and capsid protein (Cap) (3, 5, 6). Clinically, BFDV infection exhibits symptoms depending on the species and age of the bird infected, varying from sudden death to chronic progressive beak and claw deformity (7). BFDV is a hemagglutinating virus, and higher hemagglutination (HA) titer (>640 HAU/µl) in a feather or tissue sample represents clinical onset (8, 9). In the present study, we report a complete genome sequence of BFDV from a clinically infected fledgling red-capped parrot (*Purpureicephalus spurius*).

Blood and feather samples were collected from a clinically suspected fledgling red-capped parrot (identification [ID], CS15-3981/001; year of sampling, 2015; location, Wattle Grove, western Australia [32.0080°S, 116.0140°E]). Routine HA and hemagglutination inhibition (HI) tests were performed (10) as part of the PBFD diagnostic regimen, which revealed a very high level of BFDV antigen (HA titer, 1:40,960) in feather follicles, while no anti-BFDV antibody was detected in blood (HI titer, <1:20). Genomic DNA was extracted from both blood and feather samples according to established protocols (11, 12). The whole-genome sequence was amplified using the primers and PCR conditions developed in previous studies, with some modifications (13, 14). The amplified PCR products were sequenced at the Australian Genome Research Facility (AGRF), Ltd. (Sydney, Australia) using a Sanger-based AB 3730xl unit (Applied Biosystems), contigs were assembled, and the complete genome was constructed using Geneious software (version 7.1.7).

The newly amplified BFDV genome (GenBank accession no. KX449321) comprises 1,995 nucleotides (nt), with a G+C content of 53.5%. Similar to other BFDV genomes, the basic structure includes two major open reading frames (ORFs), ORF1 (nt 14 to 1000) and ORF2 (nt 1235 to 1984), containing genes encoding Rep and Cap, respectively. Preliminary BLASTn (15) analysis of the assembled whole-genome sequence revealed 99% pairwise nucleotide match with one of the Australian BFDV isolates from a red-tailed black cockatoo (*Calyptorhynchus banksii*) (GenBank accession no. KF385399) (14). Separate BLASTn and BLASTp searches for the assembled *rep* and *cap* genes also demonstrated similar results, with both having 99% sequence identity to KF385399. However, subsequent BLAST hits were mostly from BFDV genomes from different species of parrots, such as 97% nt *rep* gene identity with BDFV in African grey parrot (accession no. KF723390), while the *cap* gene showed 99% pairwise match with an isolate from a ringneck parrot (accession no. KF688549). The overall nucleotide identity of the new BFDV isolate ranges from 92 to 99% compared to the BFDV genomes available in GenBank (16). This is the first report of a BFDV genome identification in the host *P. spurius*, and it adds to the genomes obtained from *Psittaciformes* in western Australia.

### Accession number(s).

The complete genome sequence of BFDV has been deposited at GenBank under the accession no. KX449321.
